# Potential Applications of *Moringa oleifera* in Poultry Health and Production as Alternative to Antibiotics: A Review

**DOI:** 10.3390/antibiotics10121540

**Published:** 2021-12-16

**Authors:** Rifat Ullah Khan, Aamir Khan, Shabana Naz, Qudrat Ullah, Vito Laudadio, Vincenzo Tufarelli, Marco Ragni

**Affiliations:** 1 Faculty of Animal Husbandry and Veterinary Sciences, College of Veterinary Sciences, The University of Agriculture, Peshawar 25130, Pakistan; rukhan@aup.edu.pk (R.U.K.); qudrat@gmail.com (Q.U.); 2 Directorate General (Research), Livestock and Dairy Development Department, Peshawar 10320, Pakistan; aamirkhanbannuzai@gmail.com; 3 Department of Zoology, Government College University, Faisalabad 38000, Pakistan; drahabananaz@gxfu.edu.pk; 4 Department of DETO, Section of Veterinary Science and Animal Production, University of Bari ‘Aldo Moro’, Valenzano, 70010 Bari, Italy; vito.laudadio@uniba.it; 5 Department of Agro-Environmental and Territorial Science, University of Bari ‘Aldo Moro’, 70125 Bari, Italy; marco.ragni@uniba.it

**Keywords:** alternative to antibiotics, *Moringa oleifera*, poultry, nutrition, health

## Abstract

Because of developing bacterial resistance and increased public awareness of health and food safety problems, the use of antibiotics as growth promoters in the chicken industry has been outlawed. This problem has spurred the poultry industry and sector to explore for safe antibiotic alternatives and to focus on developing better long-term feed management solutions in order to improve chicken health and growth. As a result, phytogenics have developed as natural antibiotic alternatives, with a lot of potential in the poultry industry. *Moringa oleifera* has gotten a lot of attention from researchers in the recent past as a natural product with a lot of health advantages for poultry. *Moringa* is known for its antimicrobial, antioxidant, anti-inflammatory, and hypocholesterolemic properties, as well as its capacity to activate digestive enzymes in the stomach, owing to the presence of hundreds of essential ingredients. The potential influence of *M. oleifera* as a natural feed supplement on overall gut health, nutritional digestibility, blood biochemical profile, antioxidant benefits, antibacterial potential, and immunological response is emphasized in this review.

## 1. Introduction

Antibiotics are commonly administered in chicken feed and water for a variety of reasons. These drugs may be used to treat a variety of infectious disorders, mostly microbial infections. Antibiotics could also be used sub-therapeutically or prophylactically in order to avoid infections and also as a growth stimulant to improve performance and feed efficiency, however, their extensive use has resulted in antimicrobial resistance. Antibiotic resistance develops as a result of humans eating meat and eggs carrying antibiotic residues [1]. The European Union has outlawed the use of antibiotics in order to reduce their toxicity [2]. Therefore, to replace antibiotics as a growth stimulant, alternate sources are now required.

**Figure 1:**
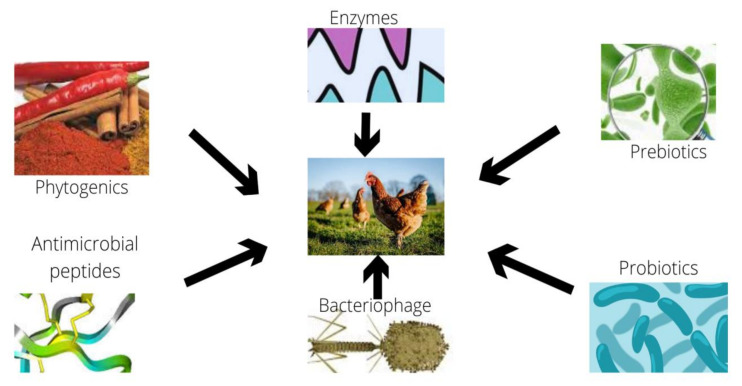
Example of some alternatives to antibiotics in poultry.

The banning of antibiotics as growth promoters, which has triggered research into alternative to antibiotics, such as probiotics [3,4,5,6,7,8,9,10], prebiotics [11,12,13,14], enzymes [15,16,17,18], organic acids [19,20], herbs [21,22,23,24,25,26,27,28,29,30,31,32,33,34,35,36] and other compounds [37,38], has been reported in poultry production (Figure 1). Researchers are developing phytogenics for usage in chickens with many advantages, such as increased feed efficiency, improved immunological status, and improved gastrointestinal health, in addition to others [39,40,41,42,43,44,45,46,47,48]. To the best of our knowledge, no review is currently available on the research outcome of *Moringa oleifera* on the growth and health aspects of poultry. Therefore, the purpose of this review was to outline the updated knowledge on *M. oleifera* on the production and health of poultry. 

*M. oleifera* is a tiny, drought-resistant deciduous tree with a height of 5 to 12 cm [49]. It has an outstanding capacity to provide nutritional, medicinal, and industrial uses to humans, as well as for animal fodder [50]. It is high in amino acids, beta-carotene, ascorbic acid, and vitamins. Because of its exceptional therapeutic and medicinal properties, it may be used as a medication to treat a variety of ailments [51]. It can also be utilized as a growth stimulant, antioxidant and antimicrobial agent [52]. The presence of caffeic acid and cinnamic acid gives it antioxidant benefits [53]. It was reported that this plant has over 92 useful components, including 36 anti-inflammatory agents, 46 antioxidants, and 18 amino acids [54]. The chemical composition of *M. oleifera* is given in Table 1. 

**Figure 2:**
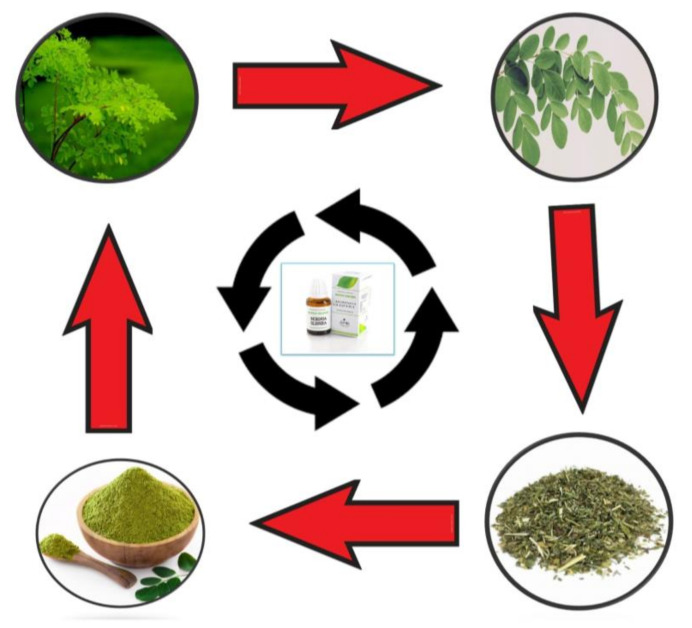
Main uses of *Moringa*: fresh, powder, as well as commercial preparations.

The leaves have a protein content of 25–27%, which is exceptional for animals. It has been estimated that the amino acid profiles of the *M. oleifera* leaves and soya-bean meal are identical [58]. The *M. oleifera* leaves contain a high protein content of 20–33% on a dry matter basis, with all of the necessary amino acids [59]. A study reported that *M. oleifera* has three times the iron content of spinach and four glasses of milk, four times the vitamin A content of carrots, and two times the protein content of milk [60]. *Moringa* is used fresh, and as powder, as well as commercial preparations (Figure 2 and Table 2).

**Figure 3:**
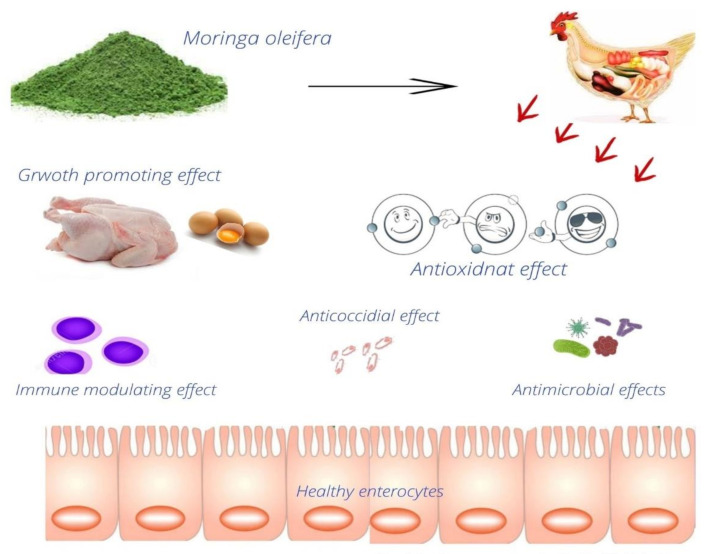
Example of some of the applications of *M. oleifera* in poultry diets.

*M. oleifera* leaf meal contains 86% dry matter, 22.5% crude fiber, 4.38% ether extract, 29.7% crude protein, 27.9% calcium, 0.26% phosphorus, and a very small quantity of tannin, according to estimates from one study [89]. It promotes immunity and has antibacterial properties [121]. *M. oleifera* contains anti-inflammatory, anticancer, antiulcer, and antioxidant effects within its various extracts and powder forms [122]. The *M. oleifera* leaf extracts show negligible or minimum toxicity levels and adverse effects in rabbits, rats, humans, and poultry [123]. Some of the examples of the uses of *Moringa* in poultry production and health are given in Figure 3. 

## 2. Feed Intake and Efficiency

Recently, a number of authors have reported an improved feed intake in response to different doses and forms of *M. oleifera* [65,66,67]. According to Zanu et al. [88], the addition of *Moringa* meal reduced feed conversion efficiency considerably. Diets containing 0%, 2.5, 5.0 and 7.5% *Moringa* leaf meal had no significant influence on broiler chicken feed intake [73]. The birds which were fed diets with 750 g/100 kg of *Moringa* leaf meal had the greatest feed-conversion ratio (FCR), which fell as the amount of *Moringa* leaf meal in the diet decreased. Paul et al. [75] concluded that the aqueous extract of the *M. oleifera* leaves reduced feed intake and enhanced the FCR in comparison with antibiotics in broiler chickens. According to El-Tazi et al. [61], using 5% *Moringa* meal in the broiler diet boosted the feed intake and feed-conversion ratio considerably. However, Khan et al. [70] reported that *M. oleifera* leaf powder had no influence on the FCR or feed intake. Khan et al. [35] reported that *M. oleifera* leaf extract had no significant influence on the FCR or the feed intake, concluding that *M. oleifera* inclusion had no negative impact on broiler feed efficiency. *M. oleifera* had a substantial influence on the feed intake and the feed-conversion ratio, according to Alabi et al. [76], with improved FCR and lower feed intake in *Moringa* extract at 90 mL/L. According to Lu et al. [69], *M. oleifera* had no significant influence on feed intake, but did have a beneficial effect on the FCR. According to Kakengi et al. [87], less than 15% *Moringa* incorporation in the diet of laying hens is advised without affecting the FCR. *M. oleifera* showed no significant influence on the feed intake and FCR, according to Ashour et al. [71]. At a level of 1–2% of the basal diet, Kwariet et al. [86] reported no significant impacts on the feed-conversion ratio for *M. oleifera* leaf meal. In contrast to the control birds, Riry et al. [77] reported that feeding Japanese quail a diet containing 5% *M. oleifera* seed meal resulted in a decrease in feed consumption. According to Elkloub et al. [78], Japanese quail which were given 0.2% *M. oleifera* leaf meal (MOLM) had the lowest feed consumption and the best feed-conversion ratio compared to those which were fed a control diet. Talukdar et al. [84] found that Japanese quail which were given 

*M. oleifera* leaf meal (of 0.25 to 0.50%) had a higher FCR than the control group that did not receive any supplementation. An improved FCR for Japanese quail which were given *M. oleifera* leaf meal at the rate of 0.1% was found by Kulkarni et al. [85]. Ahmed and El-Rayes [64] found that Japanese quail which were fed *M. oleifera* leaf meal at 3, 5, and 7 g/kg in their diet had an increased FCR and feed intake when compared to the control group that did not receive any supplementation. According to Castillo et al. [72], there was a substantial increase in the FCR for Japanese quail which were given *M. oleifera* leaf flour at the concentration of 7, 14, and 21%, but showed no significant change in feed consumption.

*M. oleifera* has a great capability of providing nutritional applications to humans and animal feedstuff, since it is an excellent source of fundamental contents, which may explain the enhanced feed intake and feed efficiency [51]. It can also be used as a growth stimulant [52]. The *M. oleifera* plant has more than 92 useful components, including 18 amino acids, according to some research [54]. According to Melesse et al. [124], broilers which were fed *M. oleifera* leaf meal showed a greater feed intake, possibly because of the enhanced bulkiness and decreased calorie content of the diet. According to Alabi et al. [125], the addition of *Moringa* meal reduced the feed consumption, which might be due to nutritional contentment. The decreased feed consumption might be due to *Moringa* having increased digestion and metabolic activities, which satisfy nutritional needs at a lower feed intake [126]. The decrease in feed intake might be attributable to a unique mix of phenolic chemicals found in *M. oleifera* leaves (zeatin, quercetin, kaempferol, and apigenin), which increase feed utilization and reduce the amount of feed required to satisfy the maintenance and production requirements [127].

However, there are discrepancies in the findings of feed intake and efficiency in *M. oleifera* supplemented birds. The reason could be due to the dose, duration, preparation of *Moringa*, in addition to strains of poultry and experimental design. 

## 3. Growth Performance and Body Weight Gain

Body weight was usually found to be improved in response to the *Moringa* treatment, although instances of no improved effects have also been reported [65,80,81,82,92]. According to Zanu et al. [88], when *Moringa* leaf meal was added to the diet, the final body weight and mean body weight decreased dramatically. When Olugbemi et al. [90] incorporated *Moringa* leaf meal into cassava-based meals, they recorded a decrease in final weight and weight gain as the level of *Moringa* leaf meal increased. Nonetheless, Du et al. [93] found no significant reduction in the growth performance of broilers which were supplemented with 0.5, 1.0, 2.0, and 3.0% *Moringa* leaf meal. The broiler diets comprising more than 5–10% leaf meals resulted in poor performance, according to Ash et al. [74]. When compared to the other experimental diets, El-Tazi et al. [61] found that the diet supplemented with 5% *Moringa* meal resulted in the most significant body weight gain. Adding *Moringa* leaf meal to the broilers’ feed boosted the weight gain in other studies [90,91,128]. The weight growth of the birds which were fed a meal containing 5% *Moringa* meal was considerably greater in the chicks given *Moringa*-based diets, which performed much better than the control group birds according to Ebenebe et al. [62]. Khan et al. [70] found that supplementing with 1.2% *M. oleifera* leaf powder raised the live body weight considerably. *M. oleifera* leaf powder had no effect on weight gain, according to Khan et al. [35]. Alabi et al. [76] reported that *M. oleifera* supplementation lowered bird growth rate and final body weight, which also had a substantial influence on the feed consumption and FCR. However, Lu et al. [69] and Hassan et al. [79] found that *Moringa* meal had a detrimental impact on broiler growth performance. The metrics of feed efficiency and body weight were enhanced by *Morigna* meal at 0.25 and 0.40%, according to Avijit Dey and Partha Sarathi De [63]. According to Elkloub et al. [78], Japanese quail which were given 0.2% *Moringa* meal gained more weight than those which were fed a control diet. Talukdar et al. [84] found a substantial increase in weight gain in Japanese quail which were fed *M. oleifera* leaf meal at the rate of 0.25 to 0.50% compared to the control group that did not receive any supplementation. *M. oleifera* leaf meal, at a concentration of 0.25 to 0.50%, can be utilized as a natural feed addition to increase the overall performance of Japanese quail. According to Kulkarni et al. [85], Japanese quail which were fed *M. oleifera* leaf meal at a rate of 0.1% gained significantly more weight than the control group. According to Ahmed and El-Rayes [64], when Japanese quail were fed *M. oleifera* leaf meal at 3, 5, and 7 g/kg of their diet, a substantial increase in weight gain was found when compared to the control group that did not receive any supplementation. Castillo et al. [72] reported a substantial reduction in weight gain in Japanese quail which were given *M. oleifera* leaf flour at 7, 14, and 21%.

*Moringa* leaves might be employed as a supplement in broiler diets to safely improve weight gain [129]. *M. oleifera* leaves have a high protein content of 25–27%, making them an excellent protein source. Makkar and Becker [58] reported that the amino acid profiles of *M. oleifera* leaves and soya-bean meal are similar. *M. oleifera* leaves contain a high protein content on a dry matter basis, with all of the necessary amino acids of excellent quality [59]. The increased protein content and reduced amount of tannins, alkaloids, and glycosides in *Moringa* are efficiently digested and may result in the improved weight gain of birds [130]. The crude extract of *M. oleifera*, similar to other herbal medications, may have digestion-enhancing qualities, stimulating the growth of beneficial bacteria while suppressing the growth of harmful microbes, and therefore, influence poultry growth and intestinal microbiota [131]. The enhanced digestibility and absorption of nutrients from the intestine in *Moringa* meal supplemented birds might explain their higher body weight [63]. According to Abdulla et al. [132], the improved weight growth of birds which were fed *Moringa* diets might be attributed to the digestion stimulatory and gastroprotective properties. According to Ambali & Furo [133], the pharmacological chemical components (cardiac glycosides, flavonoids, steroids, terpenes, saponins, and alkaloids) found in the *M. oleifera* extract may promote growth performance and body weight gain. Furthermore, carotenoids, phenolic chemicals, minerals, vitamins, alkaloids, flavonoids, and amino acids are abundant in *M. oleifera* leaves [127,134]. The high amount of vitamin C in *M. oleifera*, which can counteract the negative effects of heat stress and boost productive responses, may explain the improved performance in *Moringa* fed birds [79]. 

## 4. Carcass Traits

The leaves of *M. oleifera* are high in alpha-linoleic acid and a variety of important amino acids [55]. The high number of dietary antioxidants, such ascorbic acid and tocopherol, in *M. oleifera* may be responsible for the increase in carcass production [135]. These antioxidants reduce stress in birds while also enhancing protein absorption and digestion [136,137]. Mousa et al. [94] observed that food supplementation with *M. oleifera* increased carcass yield. El-Tazi [61] employed a variety of *M. oleifera* supplementation doses and found that a 5% supplementation in the diet boosted the carcass yield percentage when compared to the control group. Melesse et al. [95] found that increasing *M. oleifera* supplementation enhanced carcass yield. Tesfaye et al. [96] found that feeding *M. oleifera* leaf meal increased the dressing percentage, thighs, and drumstick weight significantly. Rao et al. [105] examined the varied food supplements of *M. oleifera* and concluded that *M. oleifera* had no influence on carcass yield. According to Zanu et al. [88], *M. oleifera* supplementation did not enhance dressing percentage. Carcass yield exhibited a substantial improvement in response to *M. oleifera* leaf extract [35]. According to Ahmed and El-Rayes [64], substantial increases in dressing weight were seen for Japanese quail which were fed *M. oleifera* leaf meal at rates of 3, 5, and 7 g/kg within their food when compared to the control group that did not receive any supplementation. For Japanese quail fed with *M. oleifera* leaf flour at a rate of 14%, Castillo et al. [72] found no significant influence on carcass weight and yield. The richness of *M. oleifera* in sources of carbohydrates, protein, and fiber, with minimal fat, might explain the rise in carcass weight. The availability of high pepsin and total soluble protein in *M. oleifera* leaf meal, which makes it a viable dietary protein source for simple non-ruminant animals, may explain the rise in carcass weight [128].

## 5. Egg Production and Quality

It was demonstrated that *M. oleifera* had a substantial influence on egg production and quality, as the *M. oleifera* supplemented group laid more eggs than the control group [108]. Lu et al. [69] concluded that *M. oleifera* supplementation at 15% had a significant influence on egg production, as the group treated with 15% *M. oleifera* produced less eggs. However, when the amount of *M. oleifera* increased, the egg quality in terms of yolk color and albumen height improved. When this meal was included at a 15% level in the diets, the egg production fell, while yolk rose [103]. Regardless of any antinutritional effects, the decrease in egg production might be linked to an increase in diet bulkiness as a result of rising of *Moringa* levels [97]. These findings suggest that *Moringa* meal could be added to the diets of laying hens at a rate of 15% in order to increase egg albumen quality and yolk color. Birds which were given *Moringa* at a 5% concentration increased protein retention, which improved laying performance and egg quality [138]. Ashour et al. [71] found that dietary treatments with *M. oleifera* had no effect on hatchability, fertility and egg weight, or yolk index, but dramatically improved egg production, eggshell thickness, and egg mass. Kwariet et al. [86] reported no significant impacts on egg weight of Vanaraja laying chickens when *M. oleifera* leaf meal was added to the diet at a rate of 1–2% of the baseline diet. Olugbemi et al. [90] reported that replacing sunflower seed meal with *Moringa* leaf meal (20%) in layer chicken diets resulted in a substantial drop in egg production and whole egg weight. According to Abdelnour et al. [102], *Moringa* usage of up to 10% showed no detrimental impacts on laying bird egg production, while levels larger than 10% had negative effects, probably due to the increased antinutritional components as well as low energy and protein digestibility. Talukdar et al. [84] found that quail egg quality features, such as egg weight and index of shape, albumen, and yolk, did not change; with the exception of yolk color, which exhibited a significantly greater value at the 1.00% level of *Moringa*. Yadav et al. [100] concluded that adding *M. oleifera* leaf meal into the diets of broiler and layer Japanese quail at a concentration of 1% can improve meat and egg quality and customer approval.

The richness of *M. oleifera* in sources of carbohydrates, protein, and fiber, with minimal fat, might explain the rise in egg production and egg quality. The *M. oleifera* leaves contain a high protein content of 20–33% on a dry matter basis [59]. Increased egg production may be due to the high content of carotenoids, vitamins, minerals, amino acids, alkaloids, and flavonoids in the *M. oleifera* leaves [134], as well as a rare combination of phenolic compounds (quercetin, apigenin, kaempferol, and zeatin) that are essential for growth, resulting in less feed being needed to meet the birds’ maintenance and production needs [127]. The high amount of vitamin C in *Moringa oleifera*, which can counteract the negative effects of stress and boost productive responses, may explain the improved egg production and quality in the *Moringa* supplemented birds [79]. 

## 6. Antioxidant Effects

The presence of different vitamins (E and C) and minerals (Cr, Zn, and Se), which play an essential role in the activity of oxidative enzymes, might potentially explain the decrease in oxidative stress in animals [9,10,139,140,141,142,143,144]. It was reported that *Moringa* has over 92 useful components, including 46 antioxidants, 36 anti-inflammatory agents and 18 amino acids [54,145]. According to Balami et al. [104], malondialdehyde (MDA) concentrations decreased when *Moringa* meal supplementation was increased, decreasing the stress effect on birds. Rao et al. [105] concluded that *M. oleifera* reduces lipid peroxidation and thereby reduces stress in birds. According to Karthivashan et al. [106], the *Moringa* meal supplemented group had considerably lower MDA levels than the control group. According to Cui et al. [107], *M. oleifera* supplementation groups had lower MDA than the control groups. Ratshilivha et al. [108] reported the antioxidant properties of acetone extracts from *M. oleifera*, which were tested using the DPPH technique, with the findings presented in terms of the sample concentration reducing 50% of free radical scavenging (IC50). According to Khan et al. [35], stress levels in broilers exhibited a significant response to *M. oleifera* leaf extract supplementation when compared to the antibiotic group, as seen by lower blood MDA concentrations during the finisher phase. The lower plasma MDA levels might be attributable to *M. oleifera*’s high content of antioxidants, such as tocopherol, ascorbic acids, flavonoids, and saponins. According to Elkloub et al. [78], Japanese quail which were given 0.4 and 0.6% *Moringa* meal had higher antioxidant activity than those on a control diet.

It is speculated that the presence of caffeic acid and cinnamic acid in *M. oleifera* gives it antioxidant qualities [53]. *M. oleifera* is high in dietary antioxidants, such as ascorbic acid and tocopherol, which help birds to cope with stress [136,137]. The oxidative stability of the oil derived from *M. oleifera* leaves was found to be very high, indicating the existence of natural antioxidants [103]. The flavonols, quercetin and kaempferol, in its 30-O-glycoside forms, are found in abundance in these leaves [52,97]. Flavonols are well-known chemicals that act as radical scavengers [138]. Furthermore, at equal molar concentrations, quercetin has been demonstrated to be a superior ABTS+ trap, with five times the activity of Trolox [102]. Similarly, cinnamic acid derivatives found in *M. oleifera* leaves are thought to provide various health advantages, including significant free radical scavenging characteristics, antibacterial activity, and antihyperglycemic activity [52,100,139]. According to Jung et al. [145] the gallic acid found in *M. oleifera* leaves, has antioxidant properties. The inclusion of glucosinolates, which have benzyl glycoside and hydrolyze to isothiocyanates, thiocyanates, or nitriles during enzymatic hydrolysis, boosts antioxidant activity in the *M. oleifera* leaves even more [52,146]. In lipopolysaccharide (LPS)-stimulated RAW264.7 murine macrophage cells, isothiocyanates were reported to decrease inducible nitric oxide synthase (iNOS) expression and nitric oxide generation [147]. Polyphenols were found in the aqueous extract of the *M. oleifera* leaves, according to Charoensin and Wongpoomchai [148]. *M. oleifera* leaves are also abundant in polyphenols and flavonoids, and, according to certain research also have antioxidant effects [149,150]. 

## 7. Blood Biochemistry

According to Zanu et al. [88], the *M. oleifera* supplemental food had a significant influence on triglycerides, low-density lipoprotein (LDL), very-low-density lipoprotein (VLDL), and plasma glucose concentrations, but not on blood parameters. Mahmood et al. [119] also found that *M. oieifera* supplementation lowered plasma glucose levels. The plasma protein increased with increasing levels of *Moringa* feed supplementation, according to Hassan et al. [79]. Teye et al. [151] came to the conclusion that increasing the concentration of *Moringa* meal supplementation enhanced plasma protein concentration. Apart from the mean corpuscular hemoglobin (MCH), the other hematological indices were not substantially altered by *M. oleifera* feed supplementation, according to Du et al. [93], indicating that the meals were enough to fulfill the birds’ nutrient demands. Khan et al. [35] reported that *M. oleifera* leaf extract had a substantial impact on mean blood glucose and protein concentrations, with lower serum glucose and higher serum protein concentrations during the finisher stages. According to Avijit Dey and Partha Sarathi De [63], *Moringa* supplementation induced a significant decrease in triglycerides, LDL-cholesterol, plasma total cholesterol, and a significant rise in HDL-cholesterol. *Moringa*, at a rate of 7.5%, had a detrimental influence on blood fluctuations in terms of plasma albumen and globulin, according to Onu and Aniebo [130]. *M. oleifera* had a substantial impact in layers in response to *Moringa* supplementation in terms of increased aspartate aminotransferase (AST) activity and reduced uric acid content. According to Donsbough et al. [99], 15% *Moringa* supplementation appears to have a deleterious effect on liver and renal function, as demonstrated by a greater AST activity and lower albumin and uric acid levels. Ashour et al. [71] found that blood AST and urea levels were lower in *M. oleifera* supplemented groups, while triglycerides and total cholesterol levels were also lower, with no significant effect on alanine aminotransferase (ALT), albumin, total protein, globulin, or the A/G ratio. Lu et al. [69] reported that *Moringa* leaf supplemented laying hens had lower albumen and urea concentrations than the control group. Elkloub et al. [78] concluded that different levels of *Moringa* (0.2, 0.4 and 0.6%) decreased plasma ALT and AST and cholesterol in Japanese quail, however, total protein and globulin were increased. Ahmed and El-Rayes [64] concluded that significant increased red blood cells, white blood cells, hemoglobin, calcium, and thyroid hormones were found in Japanese quail which were fed with *M. oleifera* leaf meal at the rate of 3, 5 and 7 g/kg in their diet.

Little information is available on the mechanisms through which *Moringa* influences blood biochemistry. The drop in plasma glucose concentration may be related to *M. oleifera*’s insulin-like action, which reduces gluconeogenesis and increases glucose consumption by cells [110]. *M. oleifera* has the capacity to enhance glucose absorption and use by body cells [149]. The lower plasma glucose concentrations may be linked to the regulation of hepatic gluconeogenesis and increased glucose use by body cells, which is one of the reasons *Moringa* supplementation improves dressing percentage [112,152]. The increased plasma protein concentration might be attributed to the large number of antioxidants found in *Moringa*, which have an inhibitory effect on corticosterone release, reducing protein catabolism under stressful situations and resulting in higher plasma protein concentrations [130].

## 8. Antimicrobial Activity 

*Moringa* has been demonstrated to have antibacterial properties in several investigations. Bijal and Bhumika [109] demonstrated that the ethanol, methanol, petroleum ether, and aqueous extracts of the *M. oleifera* leaves have antimicrobial activities. The findings show that solvent extracts of *M. oleifera* components (leaves, flower, pulp, and seed) were effective against *E. coli* and *S. aureus*. *M. oleifera* leaf extracts have been proposed for use in the treatment of a variety of infectious disorders, either alone or in combination with other antibiotics [153]. The experiments conducted by Patel and Mohan [111] confirmed that different *M. oleifera* extracts inhibited different bacterial strains in different ways. *Bacillus subtilis, Escherichia coli, Micrococcus luteus, Enterococcus faecalis, Klebsiella pneumoniae, Pseudomonas aeruginosa, Bacillus cereus, Serratia marcescens, Staphylococcus epidermidis, Salmonella paratyphi, Staphylococcus aureus*, and *Salmonella typhi* were among the species studied. Bichi and Shehu [154] used the agar-well diffusion technique in another recent investigation. The *M. oleifera* seed oil hexane crude extract showed strong antibacterial activity against *E. coli*, with average zones of inhibition of 17.7, 14.3, 11.3, and 9.0 mm for the 100, 75, 50, and 25% concentrations, respectively. According to Castillo et al. [72], *M. oleifera* leaf flour (14%) substantially increased the immunity in Japanese quail by preventing both Gram-positive and Gram-negative bacterial growth.

Mohamed et al. [114] investigated the antiviral potential of *M. oleifera* leaf extracts. The antiviral test of the *M. oleifera* aqueous extract at a concentration of 200 g/mL demonstrated inhibitory activity of 43.2 and 21.4% for herpes simplex virus types one and two, respectively. These findings show that *M. oleifera* can be used alone or in combination with viral medicines to treat viral infections. According to Mousa et al. [94], *M. oleifera* supplementation raised antibody titers against Newcastle disease (ND) and infectious bronchitis disease (IBD) in broiler chicks. *M. oleifera* supplementation boosted antibody titer against ND, according to Rao et al. [105]. Hassan et al. [79], on the other hand, found that increased *M. oleifera* supplementation lowered antibody titer against ND. According to Khan et al. [35], higher antibody titers against ND and infectious bronchitis (IB) were detected, indicating a substantial response to the *M. oleifera* leaf extract. The bioactive components of *M. oleifera* may increase the number of B lymphocytes, which are responsible for antibody production, resulting in a rise in antibody titer.

The mycelia growth of *Aspergillus flavus* was shown to be suppressed by *M. oleifera* (bark seed and leaf) crude extracts in a recent study by Aondo et al. [115]. *M. oleifera*’s antifungal properties can help to prevent saprophytic fungi from contaminating the culture medium. The fungi were shown to be resistant to ethyl acetate, methanolic, ethanolic, and water extracts of *M. oleifera* leaves, seeds, and bark. According to Patel and Mohan [111], distinct extracts of *M. oleifera* demonstrated varied inhibitory patterns against different fungal strains in their testing.

The enhanced leucocyte production and bursal lymphocytes might be attributed to *M. oleifera*’s antibacterial properties, which result in improved immunity [155]. The presence of diverse metabolites (chitinases, carboxylic acid, enzymes) and different lipophilic substances may possibly contribute to the increased antimicrobial action [156]. Due to the availability of natural immune modifying substances, including saponin and flavonoids, enhanced immunity was achieved by boosting lymphocytic cell growth [157]. In response to *M. oleifera* aqueous extract, the number of hematopoietic stem cells, B lymphocytes, naïve T cell expression, and proinflammatory cytokines increased [158,159]. Because of its exceptional medicinal and therapeutic properties, it may be used as a medication to prevent a variety of ailments [51,58]. It promotes immunity and has antibacterial properties [121]. *M. oleifera* exhibited antiulcer, anti-inflammatory, anticancer, and antioxidant effects in its various extract and powder forms [122].

Because the extract of *Moringa* contains a variety of compounds, all of which could trigger an immune response [155], Katanbaf et al. [160] stated that a rise in relative organ weight is seen as a sign of immunological progress. The birds in the *Moringa* supplemented groups were shown to be more energetic, fresh, and less vulnerable to prevalent diseases than the non-supplemented birds, which might be linked to the birds’ higher antioxidant status [134]. The increase in immunity might be owing to the leaves of *M. oleifera* containing a unique mix of phenolic chemicals (zeatin, quercetin, kaempferol, and apigenin) that minimize disease infestation in the gastrointestinal tract [127]. According to Lannaon [161], heightened antimicrobial activity may be due to the presence of bioceutical compounds in the *M. oleifera* plant, as well as its bacterial and immune-stimulant activities [126]. The inclusion of antibacterial elements in *Moringa* might explain the increased antimicrobial activity [162]. Furthermore, methanol and n-hexane seed extracts from *M. oleifera* and *M. stenopetala* inhibited *Salmonella typhi, Vibrio cholerae*, and *E. coli*, which are known to cause water-borne illnesses [163]. *M. oleifera* leaves have a favorable effect on improving immunological responses and intestinal health in broilers, according to Olugbemi et al. [90]. 

## 9. Anticoccidial Activity

Fadunsin and Ademola [116] found that *M. oleifera* extract had a substantial inhibitory impact on the oocyst which were shed in the feces when compared to the control group, indicating that it might be a beneficial alternative product for the treatment of avian coccidiosis in chicken production. Herbal remedies may be an option for treating coccidiosis in chickens, with the drumstick tree (*M. oleifera*) showing promise. Banna et al. [117] found that adding *M. oleifera* powder (0.5 and 1%) to broiler feed was extremely effective in reducing coccidiosis symptoms associated with experimental infection with mixed *Eimeria* species and was comparable to diclazuril (1 ppm). According to Banna et al. [117], *M. oleifera* has a strong anticoccidial activity and may be a viable option for the prevention of avian coccidiosis in chicken production. In addition to its growth-promoting effect, safety, and antibacterial action, it may be used as a preventative and curative agent for coccidia. *M. oleifera* showed no significant influence on sporulation of *Eimeria* species oocysts in hens, according to Gadelhaq et al. [118]. The mechanism of anticoccidial activity, on the other hand, is yet unknown. It is suggested that the antioxidant capabilities of *M. oleifera* may be responsible for the suppression of the oocyst shed in feces. Antioxidant chemicals, according to Allen et al. [164], are known to lessen the severity of *E. tenella* infections by reducing the degree of intestinal lipid peroxidation. 

## 10. Conclusions

Alternatives to antibiotics are used to reduce microbial populations and boost growth through a variety of mechanisms, including altering and/or inhibiting microbial growth, reducing inflammation, enhancing innate immunity, decreasing oxidative stress, and improving gut integrity. The most significant advancement in the hunt for genuine antibiotic alternatives is a better comprehension of new scientific information in order to produce unique products that can provide the benefits of antibiotics without increasing resistance. When considering phytochemicals as antibiotic alternatives, we must take into account the dosage, the differences the in active substances in plants, the toxic effects on various organs, the safety of the phytochemical leftovers in end-users, and the long-term effect of employing phytochemicals in chickens on resistance development. Although the use of phytochemicals is a relatively new topic of study, there is a need for a mechanistic approach to avoid misleading claims and to ensure safe commercialization and production. This will maximize good management and husbandry methods, with the ultimate objective of minimizing antibiotic usage in the animal industry. Understanding their mechanism of action, developing ways to standardize their effects, and refining delivery mechanisms for site-targeted distribution require further investigation. Based on the available evidence, we can conclude that including *M. oleifera* in poultry feed has positive effects on growth, blood biochemical profile, immunity, anticoccidial, antimicrobial, and antioxidant activity in the poultry industry, resulting in improved food safety, health, and economic aspects. However, it is pertinent to note that opinions are divided on the beneficial outcome of *M. oleifera* supplementation in poultry. It seems that the beneficial effects of this plant are linked with preparation, dose level, duration of supplementation and other experimental variations. In addition, little attention has been given to the mechanism of action through which *M. oleifera* produces such beneficial impacts in poultry. 

## Figures and Tables

**Table 1:** Chemical composition of *M. oleifera* (adapted from Moyo et al. [55]; Aja et al. [56]; Sobhy et al. [57].

Proximate Analysis	%	Essential Amino Acids	mg/100 g	Non-Essential Amino Acids	mg/100 g	Vitamins	mg/100 g
Carbohydrates	23.6	Leucine	94.36	Glutamic Acid	18.03	Vitamin A (β-Carotene)	16.3
Crude fiber	35.0	Lysine	69.13	Aspartic Acid	13.76	Vitamin B1 (Thiamine)	2.64
Moisture	10.0	Valine	62.34	Arginine	7.65	Vitamin B2 (Riboflavin)	20.5
Ash	10.0	Threonine	48.35	Alanine	4.93	Vitamin B3 (Nicotinic acid)	8.2
Crude protein	30.29	Isoleucine	46.98	Serine	3.13	Vitamin C (Ascorbic acid)	17.3
Crude fat	6.50	Histidine	29.56	Glycine	2.31	Vitamin E (Tocopherol acetate)	113
**Macrominerals**		**Composition (%)**		**Fatty Acids**		**Composition (%)**	
Calcium		3.65		Capric (C10)		0.07	
Potassium		1.50		Lauric (C12)		0.58	
Sulphur		0.63		Myristic (C14)		3.66	
Magnesium		0.50		Palmitic (C16)		11.76	
Phosphorus		0.30		Margaric (C17)		3.19	
Sodium		0.164		Stearic (C18)		2.13	
				Arachidic (C20)		1.61	
**Microminerals (mg/kg)**				Heneicosylic (C21)		14.41	
Zinc		31.03		Behenic (C22)		1.24	
Copper		8.25		Tricossylic (C23)		0.66	
Manganese		86.8		Lignoceric (C24)		2.91	
Iron		490					
Selenium		363					
Boron		49.93					

**Table 2:** Effects of *M. oleifera* on different parameters of poultry.

Parameter	Dose	Source	Effect	Reference
**Feed intake**	5%	MOLM	Increased	El-Tazi et al. [61]
	10%	MOLM	Increased	Ebenebe et al. [62]
	0.25 and 0.40%	MOL	Increased	Avijit Dey and Partha Sarathi De [63]
	3, 5 and 7 g/kg diet	MOLM	Increased	Ahmed and El-Rayes [64]
	2.5 and 5%	MOLM	Increased	Mikhail et al. [65]
	200 and 400 ppm	MOEO	Increased	Tekce et al. [66]
	0.3%	MOLP	Increased	Abou-Elkhair et al. [67]
	8.0%	MOLM	Improved	Egu [68]
	60–120 mL/L	MOLE	No effect	Khan et al. [35]
	5, 10 or 15%	MOL	No effect	Lu et al. [69]
	1.2%	MOLP	No effect	Khan et al. [70]
	1 g/kg	MOL and MOS and their combination	No effect	Ashour et al. [71]
	7, 14 and 21%	*M. oleifera* leaf flour	No effect	Castillo et al. [72]
	2.5, 5 and 7.5%	MOLM	No effect	Atuahene et al. [73]
	5–10%	MOLM	Decreased	Ash et al. [74]
	1%	MOLE	Decreased	Paul et al. [75]
	90 mL	MOLE	Decreased	Alabi et al. [76]
	5%	MOS	Decreased	Riry et al. [77]
	0.2%	MOLM	Decreased	Elkloub et al. [78]
	15%	MOLM	Decreased	Hassan et al. [79]
	0.75%	MOSP	Decreased	Wahab et al. [80]
	0.4 to 0.6%	Phytogenic feed mixture contained equal ratios of black cumin, *M. oleifera* and chicory seeds	Decreased	Arif et al. [81]
	90 mL/L	MOLE	Decreased	Kumar et al. [82]
	5%	MOL	Improved	Hafsa et al. [83]
**Feed efficiency**	750 g/100 kg	MOLM	Improved	Atuahene et al. [73]
	90 mL	MOLE	Improved	Alabi et al. [76]
	1%	MOLE	Improved	Paul et al. [75]
	5%	MOLM	Improved	El-Tazi et al. [61]
	5, 10 or 15%	MOL	Improved	Lu et al. [69]
	0.2%	MOLM	Improved	Elkloub et al. [78]
	0.25 to 0.50%	MOLM	Improved	Talukdar et al. [84]
	0.1%	MOLM	Improved	Kulkarni et al. [85]
	3, 5 and 7 g/kg diet	MOL	Improved	Ahmed and El-Rayes [64]
	7, 14 and 21%	*M. oleifera* leaf flour	Improved	Castillo et al. [72]
	2.5 and 5%	MOLM	Improved	Mikhail et al. [65]
	200 and 400 ppm	dietary MOEO	Improved	Tekce et al. [66]
	0.3%	*M. oleifera* seed powder	Improved	Abou-Elkhair et al. [67]
	8.00%	MOLM	Improved	Egu [68]
	0.75%	MOSP	Improved	Wahab et al. [80]
	0.4 to 0.6%	Phytogenic feed mixture contained equal ratios of black cumin, *Moringa oleifera* and chicory seeds	Improved	Arif et al. [81]
	90 mL/L	MOLE	Improved	Kumar et al. [82]
	5%	MOL	Improved	Hafsa et al. [83]
	60–120 mL/L	MOLE	No effect	Khan et al. [35]
	1 g/kg	MOL and MOS and their combination	No effect	Ashour et al. [71]
	1.2%	MOLP	No effect	Khan et al. [70]
	1–2%	MOLM	No effect	Kwariet et al. [86]
	15%	MOL	No effect	Kakengi et al. [87]
	5, 10 and 15%	MOLM	Decreased	Zanu et al. [88]
**Body weight**	5%	MOLM	Increased	El-Tazi et al. [61]
	1%	MOLM	Increased	Kakengi et al. [89]
	1%	MOLM	Increased	Olugbemi et al. [90]
	1%	MOLM	Increased	Banjo [91]
	10%	MOLM	Increased	Ebenebe et al. [62]
	1.2%	MOLP	Increased	Khan et al. [70]
	0.25 and 0.40%	MOL	Increased	Avijit Dey and Partha Sarathi De [63]
	0.2%	MOLM	Increased	Elkloub et al. [78]
	0.25 to 0.50%	MOLM	Increased	Talukdar et al. [84]
	0.1%	MOLM	Increased	Kulkarni et al. [85]
	3, 5 and 7 g/kg diet	MOLM	Increased	Ahmed and El- Rayes [64]
	2.5 and 5%	MOLM	Improved	Mikhail et al. [65]
	8.00%	MOLM	Improved	Egu [68]
	0.75%	MOSP	Improved	Wahab et al. [80]
	0.4 to 0.6%	Phytogenic feed mixture contained equal ratios of black cumin, *M. oleifera* and chicory seeds	Improved	Arif et al. [81]
	90 mL/L	MOLE	Improved	Kumar et al. [82]
	5%	MOL	Improved	Hafsa et al. [83]
	60–120 mL/L	MOLE	No effect	Khan et al. [35]
	5, 10 or 15%	MOL	No effect	Lu et al. [69]
	5%	MOL	No effect	Kilany et al. [92]
	5, 10 and 15%	MOLM	Decreased	Zanu et al. [88]
	5 and 10%	MOLM	Decreased	Olugbemi et al. [90]
	90 mL	MOLE	Decreased	Alabi et al. [76]
	7, 14, and 21%	*M. oleifera* (MOR) leaf flour	Decreased	Castillo et al. [72]
**Overall growth performance**	200 and 400 ppm	dietary MOEO	Improved	Tekce et al. [66]
	15%	MOLM	Improved	Hassan et al. [79]
	2.5 and 5%	MOLM	Improved	Mikhail et al. [65]
	8.00%	MOLM	Improved	Egu [68]
	0.75%	MOSP (*Moringa oleifera* seed powder)	Improved	Wahab et al. [80]
	0.4 to 0.6%	Phytogenic feed mixture (BMC) contained equal ratios of black cumin, *Moringa oleifera* and chicory seeds	Improved	Arif et al. [81]
	90 mL/L	MOLE	Improved	Kumar et al. [82]
	5%	MOL	Improved	Hafsa et al. [83]
	0.5, 1.0, 2.0 and 3.0%	MOLM	No effect	Du et al. [93]
	5, 10 or 15%	MOL	No effect	Lu et al. [69]
	5–10%	MOLM	Decreased	Ash et al. [71]
	10, 15, 20 and 25%	MOSM	Decreased	Hassan et al. [79]
**Carcass traits: dressing pertentage**	60–120 mL/L	MOLE	Improved	Khan et al. [35]
	1.5%	*M. oleifera* dietary supplementation	Improved	Mousa et al. [94]
	5%	MOLM	Improved	El-Tazi et al. [61]
	2, 4 and 6%	*Moringa*	Improved	Melesse et al. [95]
	3, 5 and 7 g/kg	MOLM	Improved	Ahmed and El-Rayes [64]
	5%	MOLM	Improved	Mikhail et al. [65]
	1%	MOL	Improved	Hafsa et al. [83]
	5, 10, 15%	MOLM	No effect	Zanu et al. [88]
	7, 14, and 21%	MOLP	No effect	Castillo et al. [72]
	5–20%	MOL	Decreased	Tesfaye et al. [96]
	5, 7.5 and 10%	MOLM	Decreased	Onunkwo and George [97]
**Egg production and quality**	1 g/kg	MOL and MOS and their combination	Improved	Ashour et al. [71]
	300 g	MOL	Improved	Mohammed et al. [98]
	15%	MOL	Improved	Ebenebe et al. [62]
	5%	MOL	Improved	Donsbough et al. [99]
	1%	MOLM	improved	Yadav et al. [100]
	4–6%	MOP	improved	Siti et al. [101]
	0.3%	MOP	improved	Abou-Elkhair et al. [67]
	1–2%	MOLM	No effect	Kwariet et al. [86]
	20%	MOLM	No effect	Abdelnour et al. [102]
	1%	MOLM	No effect except yolk color improved	Talukdar et al. [84]
	15%	MOL	Decreased	Lu et al. [69]
	20%	MOLM	Decreased	Olugbemi et al. [90]
	15%	MOL	Decreased	Abou-Elezz et al. [103]
**Antioxidant effects (MDA concentration)**	5%	MOL	Decreased MDA level	Balami et al. [104]
	500 and 1000 mg/kg	MOL	Decreased MDA level	Rao et al. [105]
	0.5%, 1.0%, and 1.5%	MOLM	Decreased MDA level	Karthivashan et al. [106]
	1, 2, 5, 10, and 15%	MOL	Decreased MDA level	Cui et al. [107]
	(1 g)	MOLP	Decreased MDA level	Ratshilivha et al. [108]
	60–120 mL/L	MOLE	Decreased MDA level	Khan et al. [35]
	0.4 & 0.6%	MOLM	Decreased MDA level	Elkloub et al. [78]
	90 mL/L	MOLE	Decreased MDA level	Kumar et al. [82]
	5%	MOL	Lowest TBARS level in the blood serum of broilers	Hafsa et al. [83]
	5%	MOL	No effect	Kilany et al. [92]
	15%	MOL	Increased MDA level	Lu et al. [69]
**Antibacterial activity**	10 gm of collected powdered form of leaves, flower, seed and pulp	Extracts of MOLE	Active against *E. coli* and *S. aureus*	Bijal and Bhumika [109]
	Powder (200 g) was extracted with methanol	MOLE methanolic extracts	Effective against Gram-negative bacterial strains	Dzotam et al. [110]
	Powder (200 g)	Extracts of MOLE	Effective against different bacterial strains	Patel and Mohan [111]
	Powder (200 g)	*M. oleifera* seeds oil	Effective against *E. coli*	Bichi and Shehu [112]
	14%	MOLP	Effective against both Gram-positive and Gram-negative bacteria	Castillo et al. [72]
	0.5, 1 and 5%	MOL	Decreased ileal counts of *E. coli*, *Salmonella* and *Staphylococcus.* spp. while total ileal *Lactobacillus* spp. count increased	Hafsa et al. [83]
**Antiviral activity**	10 and 20%	*M. oleifera* supplementation	Improved	Mariana et al. [113]
	200 µg/mL	MOLE	Effective against herpes simplex virus type 1 and 2	Mohamed et al. [114]
**Antibody response against NDV**	60–120 mL/L	MOLE	Improved	Khan et al. [35]
	500 and 1000 mg/kg	MOL	Improved	Rao et al. [105]
	1.5%	*M. oleifera* supplementation	Improved	Mousa et al. [94]
	15%	MOLM	Improved	Hassan et al. [79]
	0.75%	MOLP	Improved	Wahab et al. [80]
	90 mL/L	MOLE	Improved	Kumar et al. [82]
	5%	MOL	No effect	Kilany et al. [92]
	10, 15, 20 and 25%	MOSM	Decreased	Hassan et al. [79]
**Antibody response against IBV**	60–120 mL/L	MOLE	Improved	Khan et al. [35]
**Antibody response against IB**	200 µg/mL	MOLE	Effective against herpes simplex virus type 1 and 2	Mohamed et al. [114]
**Antifungal activity**	100 g/L ethanolic solvents	Extract of *M. oleifera* (Bark seed and Leaf) Crude extracts	Effective against mycelia growth of *Aspergillus flavus*	Aondo et al. [115]
		n- Hexane, ethyl acetate, methanol and distilled water Leaf, stem, flower and fruit extracts of *M. oleifera*	Effective against *Aspergillus niger, Aspergillus paracitic, Candida Albicans, Aspergillus flavus, Trichoderma harzanium, Alternata burnsi, Fusarium oxysporum*	Patel and Mohan [111]
**Anticoccidial activity**	1.0, 2.0, 3.0, 4.0 and 5.0 g/kg body weigh	Acetone extract MOLE	Inhibitory effect on oocyst shed in the faeces	Fadunsin and Ademola [116]
	0.5 and 1%	*Moringa* olifera powder	Effective against coccidial activity	Banna et al. [117]
	10% ethanolic extract	MOLP	Not Effective to inhibit or disrupt sporulation of *Eimeria* species oocysts of the chickens	Gadelhaq et al. [118]
**Blood biochemistry**	5, 10 and 15%	MOLM	Significant effect on Triglycerides, LDL, VLDL and plasma glucose concentration	Zanu et al. [88]
			Non-significant on blood parameter and Mean Corpuscular Hemoglobin (MCH) & plasma protein	
	10, 15, 20 and 25%	MOSM	Increased plasma protein	Hassan et al. [79]
	5–20%	MOLE	Increased plasma protein	Tesfaye et al. [96]
	60–120 mL/L	MOLE	Increased serum protein concentration	Khan et al. [35]
			Decreased serum glucose concentration	
	10, 30 and 50 mL/L	MOL	Decreased plasma glucose concentration	Mahmood et al. [119]
	0.5, 1.0, 2.0 and 3.0%	MOLM	Significant effect on Mean Corpuscular Hemoglobin (MCH)	Du et al. [93]
	0.25% and 0.40%	MOL	Decreased triglyceride, LDL-cholesterol, plasma total cholesterol	Avijit Dey and Partha Sarathi De [63]
			Increased HDL-cholesterol	
	15%	MOL	Higher AST activities	Donsbough et al. [99]
			Wer ALB and UA levels	
	5, 10 or 15%	MOL	Decreased albumen (ALB) and urea (UA)	Lu et al. [69]
	1 g/kg	MOLE and MOLP	Decreased blood aspartate transaminase (AST) and urea, triglycerides and total cholesterol	Ashour et al. [71]
			No significant effect on alanine aminotransferase (ALT), albumin, total protein, globulin	
	0.2, 0.4 and 0.6%	MOLM	Increased HDL, total protein and globulin	Elkloub et al. [78]
			Decreased plasma ALT & AST, plasma cholesterol & LDL.	
	8%	MOLM	Decreased glucose and cholesterol levels	Egu [68]
	0.75%	MOSP	Decreased total cholesterol and LDL	Wahab et al. [80]
	0.4 to 0.6%	Phytogenic feed mixture contained equal ratios of black cumin, *M. oleifera* and chicory seeds	Decreased total cholesterol and LDL & liver enzymes	Arif et al. [81]
	1250 ppm	MOLM	Increased serum HDL	Ajantha et al. [120]
			Decreased serum cholesterol, LDL, triglyceride and muscle cholesterol levels	
	90 mL/L	MOLE	No effect	Kumar et al. [82]
	5%	MOL	Lower WBC count and lymphocyte %age, glucose, cholesterol, triglycerides, AST and ALT concentrations.	Hafsa et al. [83]
			Higher heterophil and H/L ratio, serum protein, Ca and P levels	
	3, 5 and 7 g/kg diet	MOLM	Increased blood constituents: RBCs, Hb and PCV, WBCs, plasma total protein, albumin, Ca, HDL, GPX, GSH, SOD, TAC, IgG, and T4 hormones	Ahmed and El-Rayes [64]
			Decreased plasma cholesterol, total lipids, LDL, AST, ALT and glucose	
			No effect on phosphorus (P)	

MOL: *Moringa oleifera* leaves MOLE: *Moringa oleifera* extract, MOLM: *Moringa oleifera* meal, MOLP: *Moringa oleifera* leaf powder.
